# Time matters for resuscitation and COVID-19: double bind and duty of care

**DOI:** 10.1136/postgradmedj-2020-138509

**Published:** 2020-07-24

**Authors:** Nicola Scott, Rodric Vian Francis, Pradhib Venkatesan

**Affiliations:** Infectious Diseases, Nottingham University Hospitals NHS Trust, Nottingham, UK; Infectious Diseases, Nottingham University Hospitals NHS Trust, Nottingham, UK; Infectious Diseases, Nottingham University Hospitals NHS Trust, Nottingham, UK

## INTRODUCTION

During the coronavirus disease 2019 (COVID-19) pandemic, there has been divergent guidance on the aspects of personal protective equipment (PPE) to be worn during cardiopulmonary resuscitation (CPR). CPR involves first responders in clinical areas, often nurses, and arrest teams, comprised of junior doctors and senior nurses. They follow their instincts in responding rapidly, their training and local guidance. Public Health England (PHE) guidance advises that first responders can wear fluid-resistant surgical face masks.^1^ The Resuscitation Council UK (RCUK) guidance is that aerosol-generating procedure (AGP) level of PPE should be worn, which includes FFP3 (filtering face piece) respirators and long-sleeved gowns.^2 3^ Local Resuscitation Committees have had to grapple with divergent guidance and balance the duty of care to patients and to staff. To overcome this double bind, we consider the view that only one of these options can be followed and the counterview that an alternative, balanced approach is required.

**View**: Either follow PHE or RCUK guidance

Nurses and junior doctors require clear guidance on what to do in emergency situations. Human factors affect outcomes and it is important that the stress from uncertainty experienced by individuals is minimised.^4^ Furthermore, resuscitation is a team effort, and every member of the team should follow the same set of guidance. COVID-19 is presumed to be here for some time, and in that time staff may rotate from one hospital to another. Having a standardised approach between hospitals will facilitate the functioning of arrest teams. Ultimately, the duty of care to protect staff falls to the employing hospital trust, which has to decide which practice to follow.^5^ Both PHE and RCUK guidance consider the protection of staff and believe that the levels of PPE they advise are appropriate. There is no definitive evidence base for either view, but lines of argument are used based on available information. A pivotal point concerns the level of aerosol generation during chest compressions. A variety of respiratory particles are emitted from the respiratory tract during breathing, talking, singing, coughing and sneezing.^6^ Larger particles of 5–200 mm (droplets) gravitate downwards over limited distances. Smaller particles of ≤5 mm diameter (aerosols) can remain suspended in the air, be conveyed several metres and be inspired around the side of a fluid-resistant surgical face mask. PHE contends that the main risk is from droplets, against which fluid-resistant surgical face masks provide protection. RCUK contends that there is a risk from aerosols, their emission is increased by chest compressions and this requires the use of respirators (FFP3) that provide a tight seal around the face. There are instances of possible transmissions to healthcare workers of infections during CPR.^7–9^ Their rarity may reassure some, but their mere occurrence may lead others to enhance protection. It is difficult to be certain that any transmission was respiratory rather than through touch and fomites.

**Counterview:** An alternative path

There is also a duty of care to patients. In-hospital cardiac arrests have an 18.4% survival to hospital discharge rate.^10^ Chances of survival are reduced by significant co-morbidities, irreversible causes of cardiac arrest and a delay in the initiation of chest compressions.^11^ Delays longer than 1 min can reduce survival to discharge by half.^10 12^ The additional time taken to don PPE prior to chest compressions could therefore decrease a patient’s chance of survival. This delay would apply to all cardiac arrests, including non-infectious, non-COVID-19 patients.

**Figure 1:**
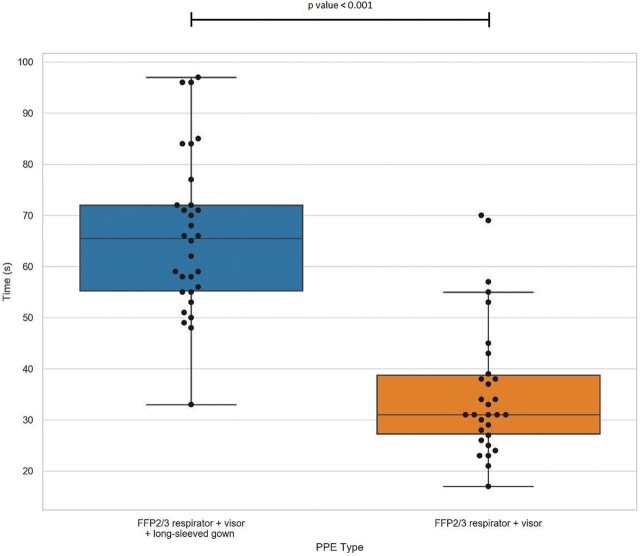
Time taken to don two versions of PPE data were analysed using JASP (JASP Team, 2020; version 0.12.0). By a two-tailed paired samples t-test, the difference was statistically significant (t(29) = 11.57, p<0.001, Cohen’s d = 2.113).

We measured the time taken to don two versions of PPE with 30 staff (16 junior doctors, 9 nurses and 5 healthcare assistants), as shown in figure 1. To start with, staff were wearing a fluid-resistant surgical face mask, plastic apron and gloves, as advised by PHE for clinical areas. Switching to an FFP3 or FFP2 respirator and donning a visor and long sleeved gown took a median of 66.2 s. Switching to an FFP3 or FFP2 respirator and visor took a median of 35.7 s. Additionally, walking to and from the furthest end of wards to the arrest trolley took on average 26 s. These times should be compared with immediate chest compressions at the bedside by staff already wearing fluid-resistant surgical face masks. Furthermore, wearing more PPE does affect the ability to perform chest compressions.^13^

There will be collective and individual views on balancing the duty of care to patients with the duty of care to staff. Rates of COVID-19 and deaths among healthcare workers understandably raise staff anxieties.^14 15^ While the majority of individuals seem to have mild disease, the healthcare workforce includes many with co-morbidities and co-factors which could worsen outlook.^16^ With patients with COVID-19, potentially infectious virus-laden aerosol or droplets could land on exposed skin, the eyes, the nose, mouth and clothing. If there were concern about skin exposure (as with Ebola), we would be covering the exposed skin on our face and neck in AGP settings. If there were concern that aerosol exposure delivers an infectious dose to our conjunctiva, we would be using tight-fitting goggles. Neither RCUK nor PHE advocate the latter two measures. The main concern for exposure to an infectious dose of virus comes from splash and droplet exposure to eyes, nose and mouth. Additionally, there is concern over inhalation of an infectious dose of aerosol particles through nose and mouth. It is recognised that chest compressions will emit aerosol, but the dispute is over quantity.^1^ Prolonged, close exposure to patients while wearing a fluid-resistant surgical face mask may afford less protection than higher levels with a respirator.^17^ Such a situation may arise if only a fluid-resistant surgical face mask is worn throughout resuscitation. There are additional risks with direct airway care and intubation.

Between two extremes, potential intermediate grades could be considered. A distinction has to be drawn between first responders and the full cardiac arrest team. The latter can don AGP level PPE on arrival, especially as members will attend to the airway. The key debate is with the former. An intermediate position would be for them to don a respirator and visor, and although they will have bare forearms, these can be washed later.

### Local guidelines for resuscitation

COVID-19 has been a divisive force in generating conflicting opinions, at a time that healthcare workers need to work together for the sake of our patients. Both local Resuscitation Committees and individuals have to consider the science, guidelines, risks and the ethical dilemmas that follow from having to balance the duty of care to patients and to staff.^18 19^ This requires a broad view and healthcare has benefited from engagement with patients and the public, the setting for this sometimes being ethics committees.^20^ But what would the public think and feel if patients who have a cardiorespiratory arrest, whether they had COVID-19 or not, had to wait 1–2 min before any resuscitation commenced? What would a healthcare worker who contracts COVID-19 in the line of duty think and feel if they were to arrest and have to wait 1–2 min before any resuscitation commenced? What would an individual or their employer think and feel if COVID-19 was acquired during CPR?

## References

[1] Public Health England . COVID-19: infection prevention and control guidance. Available https://assets.publishing.service.gov.uk/government/uploads/system/uploads/attachment_data/file/881489/COVID-19_Infection_prevention_and_control_guidance_complete.pdf (accessed 30th May 2020)

[2] Resuscitation Council UK . Resuscitation council UK statement on COVID-19 in relation to CPR and resuscitation in acute hospital settings. Available https://www.resus.org.uk/media/statements/resuscitation-council-uk-statements-on-covid-19-coronavirus-cpr-and-resuscitation/covid-healthcare/ (accessed 30th May 2020)

[3] Resuscitation Council UK . RCUK statement on PHE PPE guidance. Available https://www.resus.org.uk/media/statements/resuscitation-council-uk-statements-on-covid-19-coronavirus-cpr-and-resuscitation/statement-on-phe-ppe-guidance/ (accessed 30th May 2020)

[4] Norris EM, Lockey AS. Human factors in resuscitation teaching. Resuscitation 2012;83:423–7. 22120456 10.1016/j.resuscitation.2011.11.001

[5] Cowper A . What the law says about PPE responsibility. BMJ 2020;369:226. 10.1136/bmj.m1718 32354695

[6] Bake B, Larsson P, Ljungstrom E, et al. Exhaled particles and small airways. Respir Res 2019;20:8. 30634967 10.1186/s12931-019-0970-9 PMC6330423

[7] Couper K, Taylor-Phillips S, Grove A, et al. COVID-19 in cardiac arrest and infection risk to rescuers: a systematic review. Resuscitation 2020;151:59–66. 32325096 10.1016/j.resuscitation.2020.04.022 PMC7169929

[8] Christian MD, Loutfy M, McDonald C, et al. Possible SARS coronavirus transmission during cardiopulmonary resuscitation. Emerg Infect Dis 2004;10:28793. 10.3201/eid1002.030700 PMC3322904 15030699

[9] Nam H-S, Yeon M-Y, Park JW, et al. Healthcare worker infected with middle east respiratory syndrome during cardiopulmonary resuscitation in Korea, 2015. Epidemiol Health 2017;39:1–4. 10.4178/epih.e2017052 PMC5733382 29129042

[10] Nolan JP, Soar J, Smith GB, et al. Incidence and outcome of in-hospital cardiac arrest in the United Kingdom National Cardiac Arrest Audit. Resuscitation 2014;85:987–92. 24746785 10.1016/j.resuscitation.2014.04.002

[11] Resuscitation Council UK . Adult advanced life support. Available https://www.resus.org.uk/resuscitation-guidelines/adult-advanced-life-support/ (accessed 30th May 2020)

[12] Herlitz J, Bång A, Alsén B, et al. Characteristics and outcome among patients suffering from in hospital cardiac arrest in relation to the interval between collapse and start of CPR. Resuscitation 2002;53:21–7. 11947975 10.1016/s0300-9572(01)00485-3

[13] Malysz M, Dabrowski M, Bottiger BW, et al. Resuscitation of the patient with suspected/confirmed COVID-19 when wearing personal protective equipment: a randomized multicentre crossover simulation trial. Cardiol J. 10.5603/CJ.a2020.0068 PMC8078983 32419128

[14] Heneghan C, Oke J, Jefferson T. COVID-19 how many health care workers are infected? Available https://www.cebm.net/covid-19/covid-19-how-many-healthcare-workers-are-infected/ (accessed 30th May 2020)

[15] Cook T, Kursumovic E, Lennane S. Exclusive: deaths of NHS staff from covid-19 analysed. Health Serv J 2020. Available

[16] Coggon D, Croft P, Cullinan P, et al. Assessment of workers’ personal vulnerability to COVID-19 using “COVID-AGE”. medRxiv. 10.1093/occmed/kqaa150 PMC7454792 32761080

[17] Zhong Q, Liu YY, Luo Q, et al. Spinal anaesthesia for patients with coronavirus disease 2019 and possible transmission rates in anaesthetists: retrospective, single-centre, observational cohort study. Br J Anaesth 2020;124:670–5. 32234250 10.1016/j.bja.2020.03.007 PMC7270271

[18] Kramer DB, Lo B, Dickert NW. CPR in the COVID-19 era: an ethical framework. New Engl J Med. 10.1056/NEJMp2010758 32374958

[19] Bakewell F, Pauls MA, Migneault D. Ethical considerations of the duty to care and physician safety in the COVID-19 pandemic. CJEM. 10.1017/cem.2020.376 PMC7211799 32326998

[20] Moppett IK, Gardiner D, Harvey DJR. Guidance in an uncertain world. Br J Anaesth. 10.1016/j.bja.2020.04.003 PMC7151315 32331761

